# Coupling of colloidal rods to the dynamic order of active nematic films

**DOI:** 10.1039/d5sm01073j

**Published:** 2026-03-23

**Authors:** David P. Rivas, Louise C. Head, Jean-François Berret, Tyler N. Shendruk, Daniel H. Reich, Robert L. Leheny

**Affiliations:** a Department of Physics and Astronomy, Johns Hopkins University Baltimore MD 21218 USA leheny@jhu.edu; b School of Physics and Astronomy, The University of Edinburgh Edinburgh, EH9 3FD UK; c Université Paris Cité, CNRS, Laboratoire Matière et Systèmes Complexes 75013 Paris France

## Abstract

We report the dynamics of active nematic films and the hydrodynamic forces they generate *via* measurements on micrometer-scale magnetic rods positioned in close proximity to the films. In the absence of an external magnetic field, the rods translate with the flow of the film, and the long axes of the rods maintain parallel alignment with the film's local nematic director, even though the rods are not in direct contact with the film. The rods' translational and orientational dynamics are hydrodynamically coupled to the velocity field and its gradients in the active film. This alignment and translation facilitate measurement of correlations of the nematic director in the frame of reference of the active flow, which display a periodicity that is not present in the correlation function calculated at a fixed point in space. We identify hydrodynamic torques as the source of the rods' alignment with the time-varying local director. By applying magnetic torques *via* external fields to rotate the rods out of alignment with the director, we characterize their relaxation back toward alignment and thereby quantify the hydrodynamic torques imposed on the rods. These measurements provide insight into the flow-aligning hydrodynamic properties of active nematic films, which are thought to play a fundamental role in the nematic order.

## Abstract 2

The tracking of micrometer-scale rods in proximity to active nematic films provides a new perspective for characterizing spatio-temporal correlations in the nematic order and for measuring hydrodynamic forces generated by the films.

## Graphical abstract

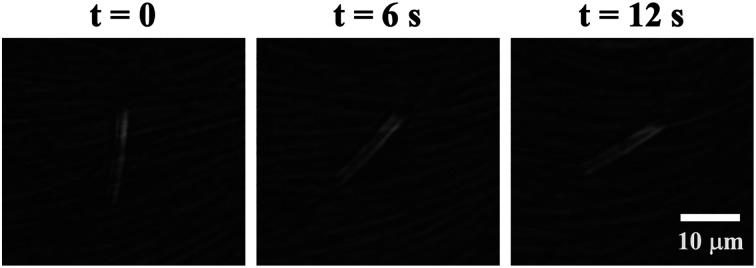

## 1 Introduction

Active nematics are a class of materials that combine the emergent properties of out-of-equilibrium active matter with the attributes of liquid crystals.^1^ Examples of active nematics include cell cultures,^2–6^ biological tissues,^7^ and agitated granular media.^8^ A well-studied realization of an engineered active nematic is a film composed of a dense packing of microtubular bundles^9^ or actin filaments^10^ driven by molecular motors at an oil–water interface. The biopolymers align with one another, endowing the films with nematic order characterized by a director field **n̂**(**r**), and slide along one another due to forces generated by the molecular motors. This sliding motion creates extensional strains in the films that drive bend instabilities in **n̂**(**r**) and ultimately lead to turbulence-like flows. The instabilities simultaneously cause creation of ±1/2 topological defect pairs in the nematic order, and the extensional strains endow the +1/2 defects with characteristic velocities, so that they act as self-propelled quasi-particles. On the other hand, −1/2 defects behave as passive non-motile quasi-particles that can be advected in the flow. The defect pairs form and annihilate at an equal rate, creating a state of dynamic equilibrium in which the film is populated with a steady-state density of defects. The ability to tune the properties of these engineered active nematics, for example the activity through ATP concentration^11,12^ or the elastic properties through biopolymer length,^10^ have made these materials fruitful testing grounds for exploring the physics of active nematics.

The flow properties of active nematic films result from a complex coupling among the hydrodynamics, the nematic elastic distortion energy, and the active stress.^13^ Experiments have investigated the dynamics of active nematic films by measuring the shearing of fluorescently marked regions of uniform nematic director, which undergo deformation due to extensional flows and diffusion, and by measuring flow velocity fields within the film by tracking fluorescently labeled microtubules.^14^ Other experiments have probed the hydrodynamic and active stresses of active nematic films^15^ and explored active dynamics in terms of turbulent mixing and topological chaos.^16–18^ Still other work has characterized the size distribution of vortices in the flow, the characteristic length scales of the nematic order, and the film speed as functions of activity, as controlled by the concentration of ATP.^12^

From the theoretical side, well-developed continuum theories successfully account for many of the effects seen experimentally.^19^ These models typically combine the nematic free energy with nematohydrodynamics that incorporates active stresses. Yeomans *et al.* have shown in theory and simulation that the hydrodynamic interactions within rod-like suspensions with extensional strains are sufficient to induce nematic order and ultimately active nematic behavior, thereby providing a physical mechanism for the ordering and dynamics in active nematics that does not invoke a nematic free energy. Specifically, the extensional strains generate dipolar flow fields that provide aligning hydrodynamic torques between adjacent rods that stabilize the nematic order on short length scales.^20,21^ Thus, these studies indicate that the nematic free energy and elasticity play a surprisingly negligible role in the properties of active nematic films, except perhaps in regions of high nematic distortion near defects. This conclusion is supported by recent data-driven approaches to infer the optimal model to describe experimental active nematic order and dynamics, which found that liquid-crystal free energy terms are negligible and that alignment dynamics are dominated by hydrodynamic coupling.^22,23^

Due to the apparent central role of hydrodynamics in active nematics, an important step in understanding these systems is to characterize their hydrodynamic forces and torques. A number of studies have investigated the response of objects within active films to the forces generated by the films.^15,24–31^ However, because these objects are embedded in the films, they are subject to elastic and steric forces whose contributions are not easily separated from hydrodynamic forces. Here, we study the dynamics of active nematic films and the hydrodynamic forces generated by the films by tracking the motion of microscale magnetic rods that are positioned in close proximity to the films. Importantly, because the rods reside in the aqueous phase near the films, as shown below, they leave the nematic order unperturbed and so are not subject to the steric or elastic forces that colloids within nematic fluids potentially experience. We observe that the rods undergo translational motion that follows the flow of the film in their vicinity while rotating to maintain alignment with the local nematic director. Taking advantage of this behavior by employing the rods as tracers, we gain a perspective on the spatial and temporal properties of the nematic order and its coupling to the flow dynamics in the film. Further, by rotating the rods away from alignment with the director using external magnetic fields and tracking their rotational dynamics after the field is removed, we characterize the hydrodynamic torques generated by velocity gradients in the active nematic.

## 2 Materials and methods

Materials to form the active nematic films were provided by the Brandeis University Materials Research Science and Engineering Center Biological Materials Facility, and the sample preparation followed the procedures described in ref. 32. The films were composed of a dense layer of fluorescent microtubule bundles adsorbed at an oil–water interface and driven by kinesin molecular motors. The samples were held between parallel glass substrates with the oil layer approximately 1–4 µm thick below the film and the aqueous layer approximately 170 µm thick above the film. As part of the film formation, magnetic rods introduced into the aqueous phase became positioned at a height approximately 5 µm above the film with their axes parallel to the film. This height, which we determined from microscopy measurements using a high-numerical-aperature objective as described in the SI, appears to be set by a thin, dilute layer of unadsorbed microtubules adjacent to the film. Additional details about the sample preparation are also provided in the SI.

The experiments employed two types of magnetic rods: ferromagnetic nickel rods and superparamagnetic rods composed of iron oxide nanoparticles in a polymer matrix. The nickel rods had an average diameter *D* of 370 nm and lengths *L* of 10 to 30 µm. The superparamagnetic rods had an average diameter of 500 nm and also lengths of 10 to 30 µm. The ferromagnetic and superparamagnetic rods hence had aspect ratios *λ* of approximately 30–80 and 20–60, respectively. The ferromagnetic rods, fabricated in-house using an electrochemical deposition technique,^33^ possessed a permanent magnetic moment parallel with their axes that was proportional to their length and was approximately 9 × 10^−13^ A m^−2^ for a 30-µm-long rod. The superparamagnetic rods possessed an internal magnetic susceptibility *χ* = 3.4 ± 0.4 and susceptibility anisotropy Δ*χ* = 2.1 ± 0.4 parallel to their axis.^34^ Details regarding the in-house fabrication and the properties of the superparamagnetic rods are described elsewhere.^34–36^ The responses of the two kinds of rods to the active nematic films were indistinguishable, indicating that the rods' specific material properties did not influence the observed dynamics described below. The concentration of rods was kept sufficiently small to ensure that interactions between rods were negligible.

**Fig. 1:**
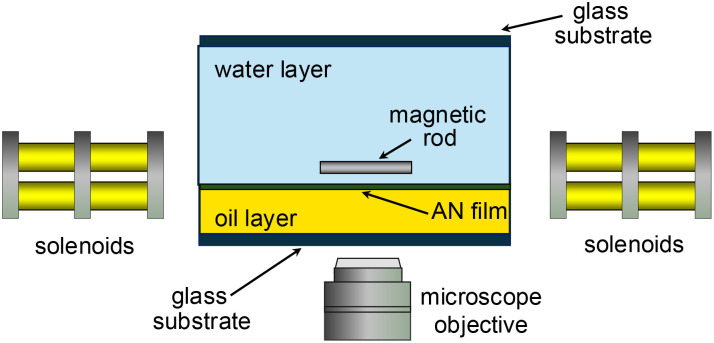
Schematic of a side view of the experiment design. A magnetic rod located approximately 5 µm above an active nematic (AN) film is subject to magnetic fields created by a set of four pairs of solenoids (two shown) mounted on an inverted fluorescence microscope.

Observations of the rods and films were performed with an inverted microscope (Nikon TE2000) using a Flare CameraLink camera (IOIndustries) operating at 1 to 3 frames per second. Both bright-field and fluorescence microscopy measurements were performed. Four pairs of solenoids mounted on the microscope^37^ generated magnetic fields of specified magnitude from 15 to 55 Gauss in arbitrary directions in the plane of the film. Fig. 1 shows a schematic of the experiment design. The time-varying nematic director field was characterized using the methods described in ref. 32 and in the SI. To track the position of a rod, the rod's center was found after a thresholding of each image was performed in which all but the darkest pixels in the image were zeroed. Then, the largest connected region of nonzero pixels was found, and all other nonzero pixels were zeroed. The center of mass (COM) of the image was then calculated and identified with the center of the rod. The grayscale intensity of the image was used as an effective mass for the computation of the COM. The orientation of the rod in each image was determined by computing the inertia tensor after thresholding. The eigenvector with the largest eigenvalue of the inertial tensor was computed, and its direction was identified as the long axis of the rod, as described elsewhere.^38^ Additional details regarding the tracking of the rods and active-nematic flow are described in the SI.

## 3 Results

**Fig. 2:**
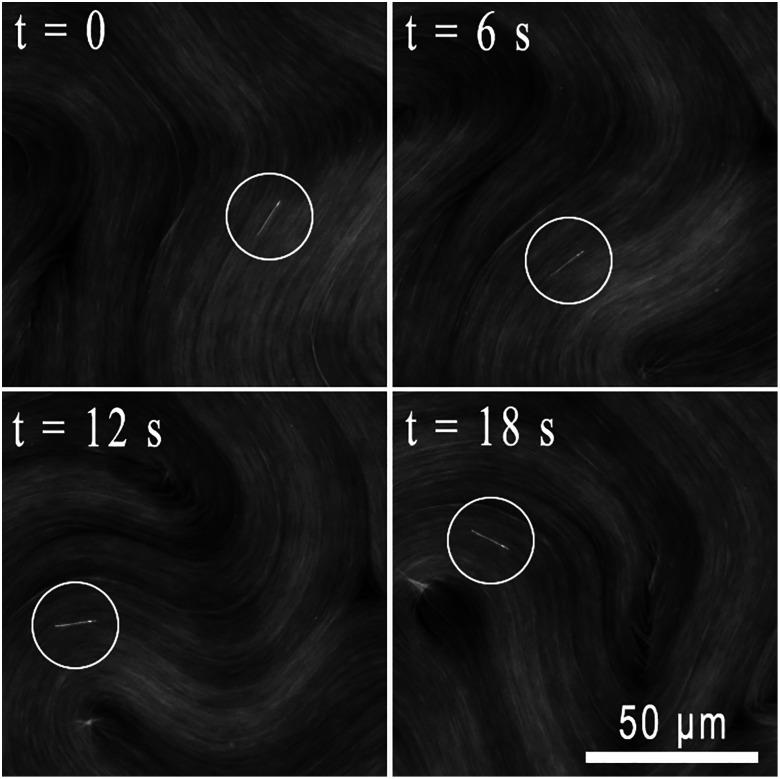
Sequence of fluorescence microscopy images showing a magnetic rod (circled) in close proximity to an active nematic film. The rod translates to follow the flow of the film while maintaining alignment with the local nematic director. The video from which these images were taken is provided in the SI (Video S1).

**Fig. 3:**
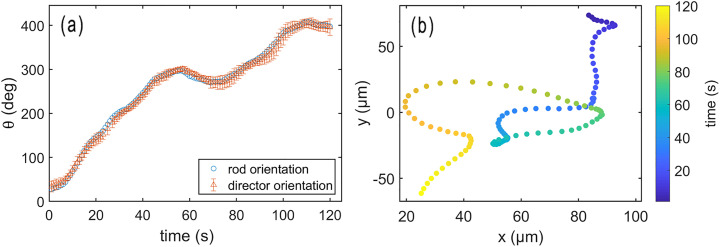
(a) The orientations of a rod and the director of an active nematic film in proximity to the rod as functions of time. The rod orientation tracks the local director as the film flows and director reorients. (b) Trajectory of the rod's center during the time interval corresponding to the orientation data in (a).

We observe that rods in proximity to the active nematic films undergo continuous translational motion seemingly with the same velocity as the film directly below them, implying a strong hydrodynamic coupling between the rods and films. Furthermore, as they translate, the rods rotate to maintain alignment with the local director. Fig. 2 shows a series of images of a rod translating and rotating along with an active nematic film in this way. The video from which the images were obtained (Video S1) and additional example videos of rods in proximity to active nematic films (Videos S2 and S3) are provided in the SI. To characterize this alignment quantitatively, we track the orientation of the rods as well as the orientation of the local director in the vicinity to the rod (averaged over an area small enough that the director orientation was approximately uniform over the analyzed region). Fig. 3(a) displays the results of such a measurement conducted over a two-minute period during which a rod followed a trajectory set by the underlying active flow shown in Fig. 3(b). The orientation of the rod axis and local director are specified in terms of the angle *θ* that each makes with horizontal axis of the microscopy images. The values of *θ* for the director orientation in Fig. 3(a) are obtained by averaging the orientation over a 26 × 26 µm^2^ region of interest (ROI) centered on the rod, and the error bars show the standard deviation in the values obtained from the sixteen 6.5 × 6.5 µm^2^ subregions of the ROI. Although the rod resides above the interface and is not in direct contact with the dense nematic film of microtubules, the orientation of the rod coincides closely with that of the underlying film's director as it translates.

We account for the rods' tendency to translate with the film and maintain alignment with the local director as arising from flows in the passive aqueous phase in which the rods reside due to the active flows within the nematic film. If the active nematic film acts as an interface that imposes a no-slip boundary condition on the water, the motion in the film must create flows in the water with velocities that match those of the film at the surface and that decay to zero at the confining glass substrate. Since the total thickness of the water is approximately 170 µm, the rods are much closer to the films than to the glass, and hence they reside in a region that has a velocity *v⃑*(**r**) essentially matching that of the nearby active film and are advected in this flow. This extrapolation of the film's active flows into the water is further illustrated by Video S4 in the SI, which shows a rod oriented perpendicular to the active nematic film by an external magnetic field that nevertheless translates along with the flows in the film.

To understand whether this interpretation for the rods' translational motion is plausible, we consider the Stokes number Stk, which is a measure of how faithfully a colloidal particle acts as a passive tracer in a flow. For the rods,1
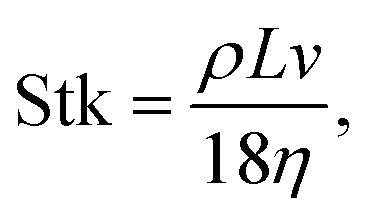
where *ρ* is the rod density, *η* ≈ 10^−3^ Pa s is the aqueous-phase viscosity, and *v* is flow velocity, which we take as the typical active flow velocities in the film, *v* ≈ 3 µm s^−1^.^32^ The largest Stokes numbers are for the longest rods composed of ferromagentic nickel for which

2 Stk ∼ 3 × 10^−5^.

This small Stokes number confirms that the rods act as translational tracer particles, advecting along with surrounding flows.

Likewise, we expect that the rotational thermal noise is negligible compared to convective rotation. The relevant dimensionless number is the rotational Peclet number,

3 Pe = *ω*_ac_*τ*_dif_,

where *ω*_ac_ is the magnitude of the vorticity in the active flows, and *τ*_dif_ is the time scale for rotational diffusion,4
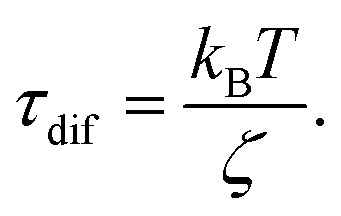
Here, *ζ* = π*ηL*^3^/3[ln(*L*/*D*) − 0.662] is the geometric rotational drag coefficient for a cylinder rotating about a short axis.^39^ Taking the typical magnitude of the vorticity to be *ω*_ac_ ≈ 0.2 s^−1^,^32^ we obtain

5 Pe ∼ 30

for the range of wire lengths and diameters in the experiment. This large Pe indicates that the time scale for rotational diffusion is far longer than the rods' reorientation times in the active flows, confirming dominance of the flows over thermal effects in reorienting the rods.

### 3.1 Correlations in nematic order

We exploit this tight hydrodynamic coupling between the rod and active nematic film to make two sets of observations. First, in this section we employ the rods as tracers to characterize correlations in the orientation of the nematic director. Second, in the next section we characterize the rotational dynamics of rods that are misaligned with the director to elucidate the hydrodynamic torques created by the active flows.

The disorderly flows of active nematic films have a complex spatio-temporal structure whose similarities to inertial turbulence have led to their description as “active turbulence”.^18,40^ Experimental and simulation efforts to describe this dynamical behavior have introduced a number of correlation functions based on the position-dependent flow velocity and director field in the films.^12,16,41^ The tendency of the rods to translate with the local flow velocity of the film while maintaining alignment with the local director provides a new perspective from which to characterize the relationship between flow and nematic order that complements other methods.^12,40,42,43^ We define a convective time auto-correlation function for the nematic director in close proximity to the rod as

6 *C*^c^_*n*_(*t*) = 2〈[**n̂**(**r**_R_(*t* + *t*′))·**n̂**(**r**_R_(*t*′))]^2^〉_*t*′_ − 1/2 = 〈cos(2[*θ*(*t* + *t*′) − *θ*(*t*′)])〉,

where the average is taken over measurement time *t*′, **r**_R_(*t*′) is the position of the rod, and the square of **n̂**·**n̂** is taken due to the symmetry of **n̂** with respect to rotations of 180°. The superscript “c” signifies that the correlation is taken between points in space given by the time-dependent position of the rod, which travels with the active flow, and hence can be considered a “convective” correlation. Perfect anti-correlation, *i.e.*, *C*^c^_*n*_ = −1, corresponds to changes in orientation of 90°, while random relative orientations average to *C*^c^_*n*_ = 0.

**Fig. 4:**
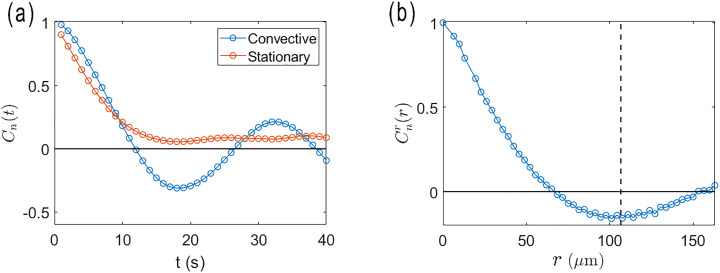
Temporal (a) and spatial (b) auto-correlation functions of the nematic director. In (a), the stationary temporal correlation function, averaged over fixed positions in space, decays monotonically, whereas the convective correlation function, averaged over a location that follows the position of a rod as it translates with the active flows, contains oscillatory features in time. In (b), the spatial correlation function also decays non-monotonically and reaches a minimum below zero. The vertical dashed line indicates the approximate spacing of +1/2 defects in the film. The data was obtained by tracking a rod of length 12 µm in a video 120 s duration capturing an area 226 × 169 µm^2^.

Fig. 4(a) displays a representative example of the convective correlation obtained from a measurement spanning 120 seconds in which a wire of length 12 µm was tracked. As the figure illustrates, the convective correlation oscillates as a function of lag time with a first minimum at *τ*_m_ = 18 s. This behavior can be contrasted with that of a stationary correlation function characterizing the time-dependent director orientation at fixed points in space,

7 *C*^s^_*n*_(*t*) = 2〈[**n̂**(**r**, *t* + *t*′)·**n̂**(**r**, *t*′)]^2^〉_*t*′,**r**_ − 1/2,

where the director is evaluated at stationary positions **r** by averaging the orientation in square windows of size ≈5.5 µm throughout the image. The superscript “s” signifies this as a “stationary” correlation function. Fig. 4(a) displays *C*^s^_*n*_(*t*) calculated from the same video from which *C*^s^_*n*_(*t*) was obtained. As the figure shows, *C*^s^_*n*_(*t*) decreases monotonically to a value near zero and is well fit by a exponential decay with a correlation time *τ*_s_ = 7.5 ± 0.8 s.

The oscillatory behavior of the convective correlation is perhaps surprising given the active turbulence in the film. In particular, the robust magnitude of *C*^c^_*n*_(*t*) over more than a full period of oscillation points to a regularity in the dynamics of the nematic director that is not apparent in other measures of the time-dependent order like *C*^s^_*n*_(*t*). At short times, *C*^s^_*n*_(*t*) and *C*^c^_*n*_(*t*) track each other, and while *C*^c^_*n*_(*t*) is not as well fit by an exponential decay as *C*^s^_*n*_(*t*), it similarly falls to 1/*e* at *t* ≈ 8 s. This similarity indicates that the characteristic time scales of both functions are set by the same dynamical properties of the film. However, the chaotic dynamics that one might infer from the exponential decay of *C*^s^_*n*_(*t*) belies a more persistent memory in the dynamic nematic order that tracking the rod co-moving with the flow reveals.

Insight into the structure of *C*^c^_*n*_(*t*) and *C*^s^_*n*_(*t*) can be found in examining the equal-time spatial correlations in director field characterized by

8 *C*^r^_*n*_(*r*) = 〈cos(2(*θ*(*r*) − *θ*(0)))〉,

where the superscript “r” specifies this is a spatial correlation dependent on *r*, the distance between points. Fig. 4(b) shows *C*^r^_*n*_(*r*) obtained from the same video as the time correlations shown in Fig. 4(a). The spatial correlations show an initial decay that can be fit to exponential function to give a correlation length of *ξ* = 43 ± 5 µm. The correlation does not decay monotonically, however, and reaches a minimum below zero at approximately *d*_m_ = 101 µm. For comparison, the approximate defect–defect separation of the positive defects in the film is 107 µm. Based on several trials, we find the defect–defect separation is approximately equal to the location of the minimum in *C*^r^_*n*_(*r*). Dividing the characteristic distances, *ξ* and *d*_m_, by the stationary exponential decay time, *τ*_s_ and the time of the convective correlation minimum *τ*_m_, respectively, gives characteristic speeds, *v*_s_ = *ξ*/*τ*_s_ ≈ 5.7 µm s^−1^ and *v*_c_ = *d*_m_/*τ*_m_ ≈ 5.6 µm s^−1^. As a comparison, we measure the average speed of the +1/2 defects to be 6.5 µm s^−1^, the average speed of the −1/2 defects to be 2.9 µm s^−1^, and the average speed of the rod to be 2.9 µm s^−1^. Therefore, we interpret the time scales associated with the features in Fig. 4(a) with the time required for the +1/2 defects to move characteristic distances.

### 3.2 Hydrodynamic torques on rods

**Fig. 5:**
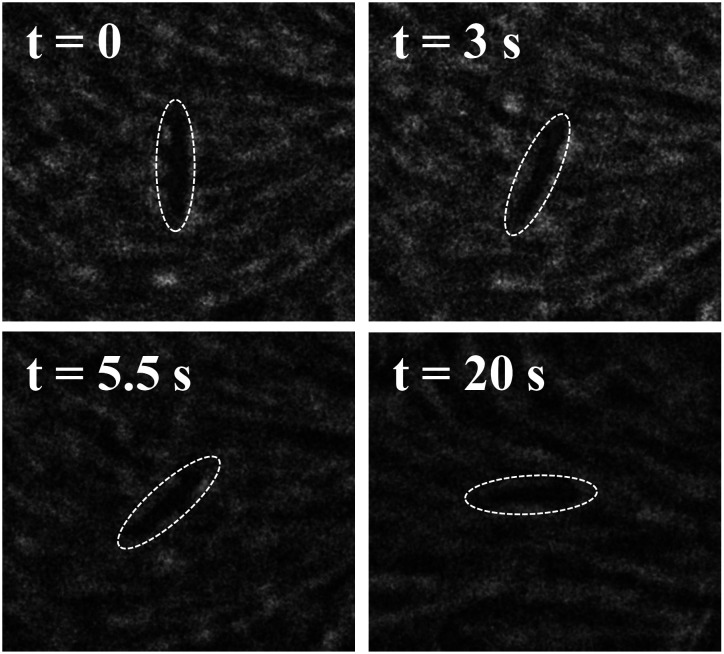
(a) Sequence of micrographs of a superparamagnetic rod, length 29 µm, at several times following removal of an applied 15 G field that initially rotated the rod to an angle Δ*θ* ≈ 80 degrees with respect to the local director. The bright-field images have been processed to accentuate the nematic texture for easier identification of the local nematic director. The video from which these images were taken is provided in the SI (Video S5).

**Fig. 6:**
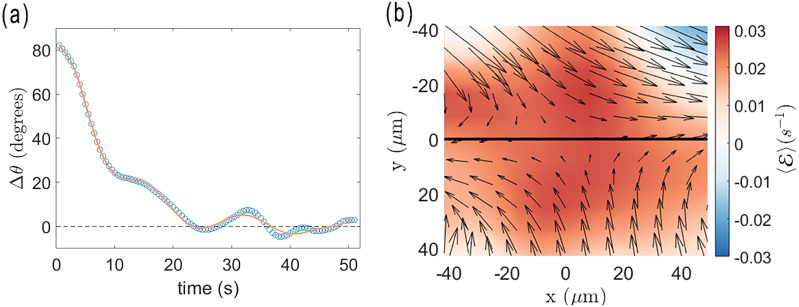
(a) The angle of a magnetic rod, length 29 µm, with respect to the local director as a function of time following removal of an applied 15 G field. The line shows the results of a fit using an 11th-order polynomial to the experimentally measured Δ*θ*(*t*). (b) A color map of the time average of the extensional rate ℰ in a frame of reference in which the rod is at the center and the director is along the *x*-axis. The time-averaged velocity field is shown by the arrows.

As described in the Introduction, hydrodynamic forces are believed theoretically to play a dominant role in promoting nematic order in active nematic films, and the persistent alignment of the rods with the local director provides an avenue to study these effects experimentally. To investigate the mechanism behind the alignment of the rods with the local director, we apply magnetic fields to rotate the rods away from the director and track the rods' orientations following removal of the field as the rods rotate back into alignment. Fig. 5 shows a series of images from such a measurement using a superparamagnetic rod of length 29 µm that was initially rotated approximately 80 degrees away from the director by a 15 G field. The video from which the images were obtained (Video S5) and additional example videos of rods rotating toward the director after misalignment (Videos S6–S9) are provided in SI. Fig. 6(a) shows the angle Δ*θ* between the rod axis and the local director as a function of time after removal of the field. The rod rotates monotonically toward the director and aligns after about 25 seconds; however, the rate of decrease of Δ*θ* is unsteady, because the director itself is reorienting during the measurement as a consequence of the flows in the active nematic.

Notably, we find the rods create no measurable distortion of the local director field as a consequence of their field-driven misalignment, as Fig. 5 and the videos in the SI illustrate. Thus, unlike the case of rod- or disk-shaped colloids in thermotropic liquid crystals^44–49^ that similarly orient with respect to the local director, the torque aligning the rod with director of the active nematic film appears not to result from the nematic elasticity. Instead, we identify the torques that align the rods with the director as resulting from the hydrodynamic coupling between the rods and the extensile and shear flows in the surrounding water created by gradients in the velocity of the active nematic film beneath it.

The temporally and spatially varying velocity field in active nematic films and their relationship to the nematic director have been characterized from a number of perspectives.^12,42,50^ As an illustration of the extensional and shear strains in the flows, Fig. 6(b) shows the time-averaged velocity field of the active nematic film in the vicinity of the rod during the measurement of the rod's rotation from Fig. 6(a). The velocities are shown in a reference frame in which the center of the rod is at the center of the plot and the local director is along the horizontal. Specifically, the video frames used in the analysis are centered at the center of the rod and rotated such that the director is oriented along the *x*-axis. As described in the SI, particle-image-velocimetry (PIV) measurements tracking features in the active nematic film determined the velocity field between adjacent frames, and the time average of the velocity at each position is obtained. As can be seen from the figure, the time-average flows have an extensile component where the film flows outward along the director and inward perpendicular to the director as a consequence of the activity. We quantify the extension in the flow by the extensional rate, 
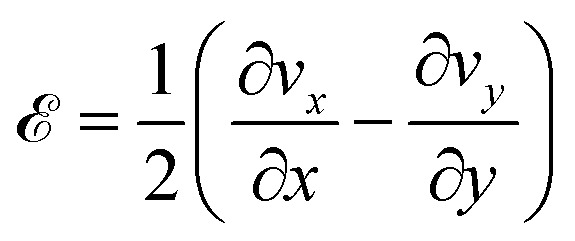
, whose time average is shown by the color map in Fig. 6(b). As expected, the time-average extension rate is greater than zero over nearly the entire field of view. The flow also has a shear component that is most evident in the top portion of the image and that can be quantified in terms of the shear rate, 
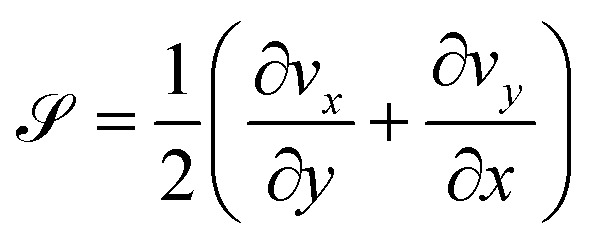
.

Taking the time average of the flow field is justified because the rod is expect to rapidly reorient if it is misaligned but to stay aligned without tumbling for longer than the flow persists. This can be seen through the dimensionless number that we denote Je, which compares the rate of change of the active turbulent flows to the rate of change of the rod orientation. If the active flows change suddenly and the rod orientation responds slowly, then the orientation of the rod may lag behind the velocity gradients of the flows. To estimate the rate of change of the flows, we use the characteristic vorticity of the active turbulence *ω*_ac_, and to estimate the time required for rods to reorient in the flow, we use the period of Jeffrey orbits, 
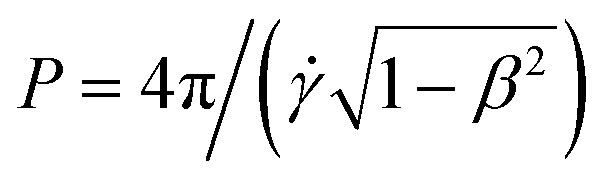
, where *β* = (*λ*^2^ − 1)/(*λ*^2^ + 1) for a rod with aspect ratio *λ*. The dimensionless ratio is then

9 Je = *ω*_ac_*P*.

The shear rate *γ̇* must be comparable to *ω*_ac_, hence we obtain10
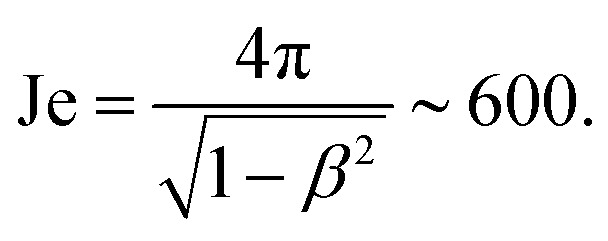
 This large value of Je indicates that the tumbling period is longer than the active time scale. Thus, the rods that are misaligned with the flow rapidly rotate but rods that are aligned with the flow remain aligned without tumbling for times much longer than the coherent flow persists.

The combination of extensile and shear flows like those in Fig. 6(b) can drive the rotation of the rods toward alignment with the nematic director. To understand these effects quantitatively, we model the hydrodynamic torque on a rod arising from the coupling with the surrounding flow field, where we assume the fluid around the rod moves at the same local velocity as the film beneath. The torque d**Γ** on a segment of rod of length d*r* due to the fluid is d**Γ** = *γ***r**′ × **v**d*r*, where **r**′ is the vector from the center of the rod to a point along its axis, **v** is the velocity of the film relative to the velocity of the rod at that point, and *γ* is a drag coefficient that depends on viscosity. The total torque **Γ** on a rod can then be found by integrating these infinitesimal contributions along the length of the rod.^51^

**Fig. 7:**
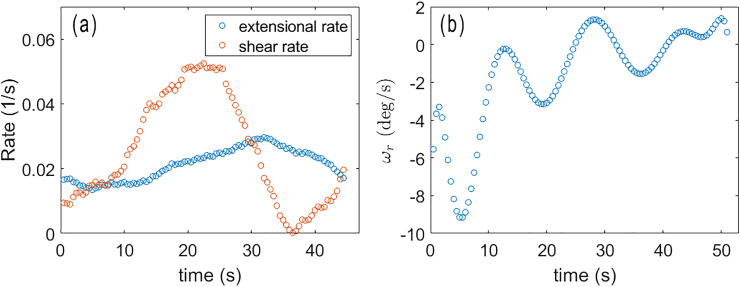
(a) The time-dependent extensional strain rate 〈ℰ〉 and shear rate 〈𝒮〉 in vicinity of the rod whose angle relative to the director is shown in Fig. 6. (b) The rotation rate of the rod relative to the director, as determined by fitting Δ*θ* to a polynomial and taking the derivative.

Because the PIV analysis does not provide a map of the film velocity with adequate spatial and temporal resolution to determine the time-dependent torque directly, we decompose the flow field into three components – extension, shear, and solid rotation – and analyze the videos in the frame of reference illustrated in Fig. 6(b). In this reference frame, the rate of solid rotation is given simply by the time derivative of Δ*θ*, *ω*_r_ = dΔ*θ*/d*t*, and the torque on a rod due to the solid rotation is 
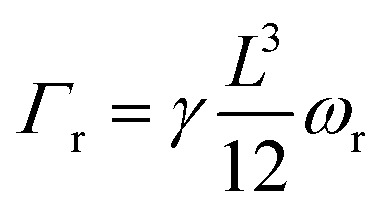
, where *L* is the length of the rod. The magnitudes of the extensional and shear rates are found by averaging over a small region of interest around a rod. Specifically, we obtain spatial averages of ℰ and 𝒮 by averaging over a cropped region of dimensions approximately twice the rod length. In Fig. 6(b), the average is taken over a region approximately 60 × 60 µm^2^ around the 29-µm-long rod. We find this size to be a good compromise between capturing the region whose flows most influence the rod's motion, while including data with adequate statistics and mitigating the influence of the rod's motion on the PIV analysis. Fig. 7(a) shows results for 〈ℰ〉 as a function of time obtained in this way.

To obtain adequate statistics for 〈𝒮〉, further simplifications are required. Although, in general, the shear includes a component from velocities perpendicular to *n̂*, empirically we observe shear primarily in flow parallel to *n̂*, perhaps reflecting an anisotropy in the film's shear viscosity as observed commonly in nematics.^52^ Hence, we focus on these contributions by averaging the velocity along *n̂* to obtain a velocity as a function of distance from the center of the rod perpendicular to *n̂*. A third-degree polynomial is fit to the data, and the derivative is found at the center of the rod to determine the shear rate. Fig. 7(a) shows the resulting 〈𝒮〉 as a function of time for the example experimental trial (Fig. 6).

The torque on a rod due to the extensional flow is found by integrating d*Γ* along the length of the rod using the velocity profile of an extensional flow field, leading to 
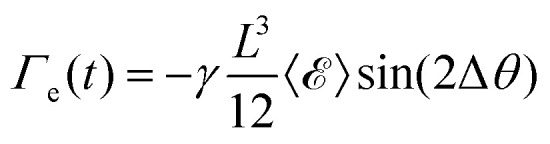
. This torque is maximized at Δ*θ* = 45° and zero at Δ*θ* = 0° and 90°. By similar analysis, the torque on a rod due to the shear flow field is 
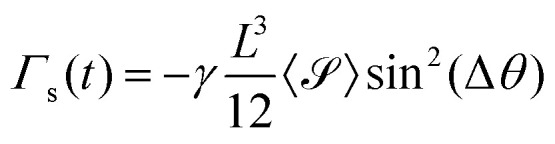
. In contrast, this torque is maximized at Δ*θ* = 90° and zero at Δ*θ* = 0°. For small values of Δ*θ*, 
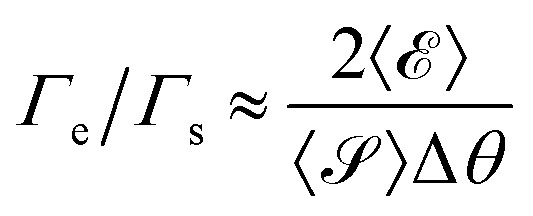
. We find that 〈ℰ〉(*t*) and 〈𝒮〉(*t*) are typically of similar magnitude, as Fig. 7(a) illustrates; therefore, at small Δ*θ*, the torque from the extensional flows dominates. Further, because 〈ℰ〉 is invariably positive due to the extensional strains inherent in the active nematic film, *Γ*_e_ acts as a restoring torque to rotate the rod back into alignment with the director for small misalignments, explaining the tendency of rods that are aligned with the director to remain aligned, as in Fig. 2.

**Fig. 8:**
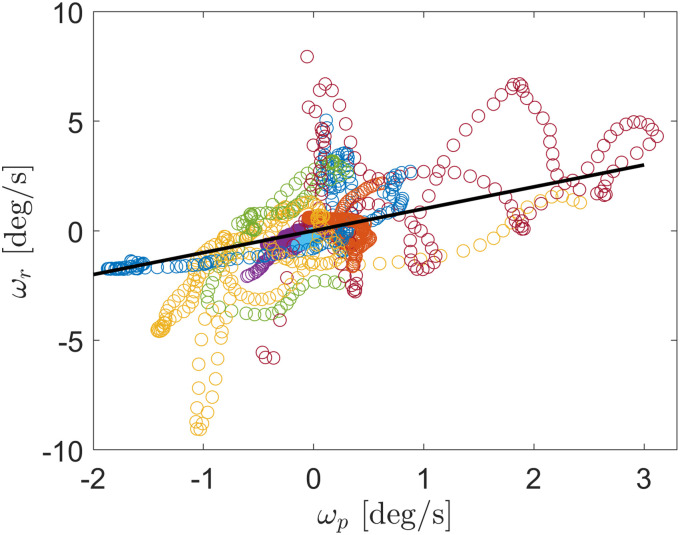
The rotation rate of rods relative to the nematic director, *ω*_r_ = dΔ*θ*/d*t*, during their angular relaxation toward the director following misalignment by an applied magnetic field. The rates are plotted against the predicted rate based upon the shear and extensional flows near the rod, *ω*_p_. The differently colored data points represent ten different measurements performed on ten different rods. The solid line shows a linear relationship with a slope of +1, which matches the predicted relationship between the rotation rates (eqn (12)) with no adjustable parameters.

When a rod is misaligned with the director by larger values of Δ*θ*, it experiences the hydrodynamic torques due to all three components of the flow: *Γ*_r_, *Γ*_e_, and *Γ*_s_. Since the system is at low Reynolds number, if no other torques act on the rod (such as from nematic elasticity), then these sum to zero,11
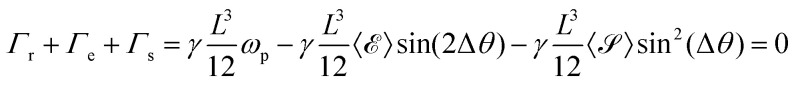
or,

12 *ω*_p_ = 〈𝒮〉sin^2^(Δ*θ*) + 〈ℰ〉sin(2Δ*θ*),

where the subscript “p” marks this as the predicted rotation rate, as opposed to the measured *ω*_r_(*t*). To test this prediction, we find *ω*_r_(*t*) by fitting an 11th-order polynomial to the experimentally measured Δ*θ*(*t*) and taking its derivative. An example of such a fit is shown in Fig. 6(a), and the resulting *ω*_r_(*t*) is shown in Fig. 7(b). Fig. 8 shows results for *ω*_r_ from a set of measurements plotted against the prediction in eqn (12). The different colors of the data points correspond to ten different measurements on misaligned rods rotating back toward the director. A linear relationship with a slope of 1, which would indicate that the prediction (eqn (12)) matches the measurements without any adjustable parameters, is shown by the solid line. As can be the case when taking numerical derivatives of data, the results in Fig. 8 are quite noisy, so one must be cautious in reaching any quantitative conclusion. However, the predicted linear relationship seems to be confirmed, strongly suggesting the predominance of hydrodynamic torques in aligning the rods with the director.

## 4 Discussion and conclusion

This investigation of the translational and rotational dynamics of rods in proximity to an active nematic film has illuminated several features of the active nematic. First, the rod dynamics have facilitated characterization of the time autocorrelation function of the nematic director in a reference frame co-moving with the local film velocity, which shows features distinct from the time autocorrelation in the lab frame. While the monotonic decay of the director time autocorrelations in the stationary lab frame is unsurprising given the turbulent-like flows within the film that act to decorrelate the director, the oscillations in the convective autocorrelation function in the co-moving frame indicate that the director undergoes a more regular and predictable periodic motion in this frame. The time scale of this periodicity can be associated with the approximate time required for the +1/2 defects within the film to move a typical defect–defect spacing.

A second major finding of this investigation is the observation that the alignment of the rods with the nematic director is driven by hydrodynamic torques arising from shear and extensional strains in the flow of the films and the quantitative characterization of these torques. This behavior hence demonstrates the tendency of the hydrodynamic forces in active suspensions of filaments with extensional strains to drive such alignment. Notably, the alignment of the rods with the nematic director occurs in the absense of any observable coupling between the rods and the nematic elasticity. The hydrodynamic forces experienced by the rods are not unlike the hydrodynamic forces experienced by the constituent microtubule bundles themselves, highlighting their role in aligning the microtubules in the film. As such, the results provide experimental support for recent theoretical work^20,21^ that finds hydrodynamic forces rather than liquid crystal free energy are predominant in promoting nematic order in suspensions of active rods. Thus, the results lend insight into the fundamental, dynamic mechanism of formation of active nematic systems.

## Conflicts of interest

There are no conflicts to declare.

## Supplementary Material

SM-022-D5SM01073J-s001

SM-022-D5SM01073J-s002

SM-022-D5SM01073J-s003

SM-022-D5SM01073J-s004

SM-022-D5SM01073J-s005

SM-022-D5SM01073J-s006

SM-022-D5SM01073J-s007

SM-022-D5SM01073J-s008

SM-022-D5SM01073J-s009

SM-022-D5SM01073J-s010

## Data Availability

Supplementary information (SI) is available. See DOI: https://doi.org/10.1039/d5sm01073j. Additional data sets generated during the study are available from the corresponding author on request.
