# Supplementary material for: Coupling of colloidal rods to the dynamic order of active nematic films
Source: Soft Matter. 2026 Mar 23;22(15):2823–32. doi: 10.1039/d5sm01073j (PMC13044720; doi:10.1039/d5sm01073j)
Supplement: SM-022-D5SM01073J-s001 [file SM-022-D5SM01073J-s001.pdf]

Supplementary Information (SI) for Soft Matter.
This journal is © The Royal Society of Chemistry 2026

# Supplementary Information for "Coupling of colloidal rods to the dynamic order of active nematic films"

David P. Rivas,[1] Louise C. Head,[2] Jean-François Berret,[3]
Tyler N. Shendruk,[2] Daniel H. Reich,[1] and Robert L. Leheny[1]

[1] *Department of Physics and Astronomy, Johns Hopkins University, Baltimore, MD, 21218, USA*
[2] *School of Physics and Astronomy, The University of Edinburgh, Edinburgh, UK*
[3] *Université Paris Cité, CNRS, Laboratoire Matière et systèmes complexes, 75013 Paris, France*

## I. LIST OF SUPPLEMENTARY VIDEOS

**VideoS1:** Microscopy video showing a rod in proximity to an active nematic film. The acquisition frame rate was 1 fps. The images shown in Fig. 2 are taken from this video.
**VideoS2:** A second example microscopy video showing a rod in proximity to an active nematic film. The acquisition frame rate was 2 fps.
**VideoS3:** A third example miscroscopy video showing a rod in proximity to an active nematic film. The acquisition frame rate was 1 fps.
**VideoS4:** Microscopy video showing a magnetic rod in proximity to an active nematic film and oriented roughly perpendicular to the film by a hand-held magnet. The acquisition frame rate was 1 fps. The rod translates with the underlying film, illustrating the influence of the hydrodynamic forces in the aqueous phase imposed by the nearby film.
**VideoS5:** Microscopy video showing a magnetic rod in proximity to an active nematic film rotating to re-align with the local director. The acquisition frame rate was 2 fps. Before the start of the video, the rod was misaligned with the director by a magnetic field that was then removed. The image contrast has been enhanced to show the nematic texture more clearly, as described below in Sec. IV. The images shown in Fig. 5 are taken from this video.
**VideoS6:** A second example microscopy video showing a magnetic rod in proximity to an active nematic film rotating to re-align with the local director. The acquisition frame rate was 1 fps. Before the start of the video, the rod was misaligned with the director by a magnetic field that was then removed. The image contrast has been enhanced to show the nematic texture more clearly, as described below in Sec. IV.
**VideoS7:** A third example microscopy video showing a magnetic rod in proximity to an active nematic film rotating to re-align with the local director. The acquisition frame rate was 2 fps. Before the start of the video, the rod was misaligned with the director by a magnetic field that was then removed. The image contrast has been enhanced to show the nematic texture more clearly, as described below in Sec. IV.
**VideoS8:** A fourth example microscopy video showing a magnetic rod in proximity to an active nematic film rotating to re-align with the local director. The acquisition frame rate was 2 fps. Before the start of the video, the rod was misaligned with the director by a magnetic field that was then removed. The image contrast has been enhanced to show the nematic texture more clearly, as described below in Sec. IV.
**VideoS9:** A fifth example microscopy video showing a magnetic rod in proximity to an active nematic film rotating to re-align with the local director. The acquisition frame rate was 1 fps. Before the start of the video, the rod was misaligned with the director by a magnetic field that was then removed. The image contrast has been enhanced to show the nematic texture more clearly, as described below in Sec. IV.

## II. SAMPLE PREPARATION

### A. Active Gel Fabrication

The active gel was fabricated by combining appropriate quantities of microtubules, ATP, and a premix solution that contained kinesin clusters, anti-bleaching agents, polyethylene glycol as a depletant, an ATP regeneration system, and buffer, as described in Ref. [1]. The microtubules and premix were provided by the Biological Materials Facility at Brandeis University. The final concentration of polyethylene glycol was 0.8% (w/v) and the final concentration of ATP was typically 1.4 mM. The microtubules were labeled with AlexaFluor 647 to enable video tracking of moving bundles using fluorescence microscopy. Prior to formation of an active nematic film, a dilute quantity of magentic rods was added to the mixture.

### B. Sample Cell Fabrication

Sample cells were fabricated from two glass slides approximately 1" × 0.5" that were first cleaned in hot water containing 1% Hellmanex (Hellma) solution and sonicated for ∼10 minutes. One slide was made hydrophilic by coating with polyacrylamide, and the other slide was hydrophobically treated using Aquapel (Pittsburgh Glass Works). The hydrophilic treatment involved first sonicating the cleaned glass in ethanol for ten minutes, then rinsing with deionized (DI) water and sonicating in 0.1

M KOH, also for approximately ten minutes. A solution of DI water and 2% w/v acrylamide was placed under vacuum for at least 15 minutes prior to adding 0.035% tetramethylethylenediamine and 0.7 g/L ammonium persulfate. The slides were soaked in a solution of 98.5% ethanol, 1% acetic acid, and 0.5% 3-(trimethoxysilyl) propylmethacrylate for roughly 15 minutes, rinsed again, and then soaked overnight in the acrylamide solution. The slides were rinsed in DI water and dried prior to use. The hydrophobic treatment involved coating the glass in Aquapel for approximately one minute then rinsing with DI water and drying. Thin strips of double-sided tape were placed on the surface of the hydrophobic slides to create two channels of approximate dimensions 2 cm $\times$ 0.2 cm in which two active nematic films were created. The hydrophilic slides were then laid on top and pressed down onto the tape.

Fluorinated oil (HFE 7500, RAN Biotechnologies) was pipetted into a channel via capillary action. The active gel containing a dilute dispersion of magnetic rods was immediately pipetted into the same channel displacing the oil except for a thin layer adjacent to the hydrophobic surface. The sides of the cell were then sealed with Norland Optical Adhesive 81 (Norland Products), and the cell was centrifuged for 10-15 minutes at 800 rpm (103 g) driving the microtubules to the oil-water interface and leading to formation at the interface of a dense film of microtubule bundles with nematic order. The centrifugation also caused the rods to go to the oil-water interface; however, they did not embed in the film but remained in the aqueous phase at a height $d = 3$ to 8 $\mu$m above the film and oriented with the rod axes parallel to the film, as depicted schematically in Fig. 1(a).

## III. VIDEO MICROSCOPY DETAILS

Observations of the rods and film were made with an inverted microscope (Nikon TE2000) and a 20X objective (Nikon, CFI LWD DL 20X). Rods selected for measurements were sufficiently isolated from other rods that the influence of other rods could be safely ignored. Videos were captured using a Flare CameraLink (IOIndustries) camera with a frame rate of 1 to 3 frames per second. An exposure time of one over the frame rate was typically used for fluorescent imaging. Image analysis was conducted using custom Python scripts, particle imaging velocimetry (PIV) in Matlab, and ImageJ.

Magnetic fields were applied using an array of solenoids with soft magnetic cores that was mounted on the microscope stage. Two sets of four solenoids positioned symmetrically above and below the sample position allowed for application of fields in arbitrary direction. The feedback-regulated current to the coils was computer controlled using custom Labview software, as described elsewhere [2]. In the experiments, magnetic fields were applied in the plane of the film at orientations that caused the rods to rotate through large angles from the local director. The magnitudes of the fields employed in the experiments ranged from 15 to 55 G, which were adequate to cause the rods to re-orient until they were essentially parallel to the field. After the rods completed their rotation to the field, the field was set to zero, and the orientations of the rods were tracked as they relaxed back to parallel with the local director. The change in field occurred on a millisecond time scale, much faster than the duration of the rods' rotations; therefore, any transient effects associated with the change in field could be ignored.

Measurements of the heights of the rods relative to the films involved adjusting a motorized z-stage on the microscope between positions at which the film and the disk were in focus. An objective with lower depth of field (Nikon, CFI Plan Fluor ELWD 40X) was employed in these measurements for more precise determination of the height, and we estimate the precision of the height measurements to be $\pm 2$ $\mu$m.

## IV. IMAGE ANALYSIS OF THE FLOW AND NEMATIC DIRECTOR FIELD IN THE ACTIVE NEMATIC FILMS

To quantify the velocity field in the active nematic films as shown in Fig. 6(b), we employed particle image velocimetry (PIV) techniques [3] to measure the displacement of microtubule bundles in subsections of the film between adjacent video frames. These results were binned and averaged by position relative to the rod as determined by the mean location of the rod center in the image pair to produce the results shown in Fig. 6(b).

The director orientation was computed using Matlab code based on the Fibriltool algorithm [4] and described previously [5]. Images were first processed by subtracting a median value from a subset of images in the video to maintain more uniform intensity across the image and remove the effects of dust or other artifacts. The code then computed the gradient in the image intensity, based on differences in intensity of neighboring pixels. Blurring was applied to the image to reduce the effects of noise and pixelation. Then, a normalized vector perpendicular to the gradient was computed as $\mathbf{t} = (t_x, t_y) = (\frac{dI}{dy}, -\frac{dI}{dx})/\sqrt{(\frac{dI}{dx})^2 + (\frac{dI}{dy})^2}$, where $I$ is the image intensity. From this, the local nematic tensor was computed: $\mathbf{n} = \mathbf{t} \otimes \mathbf{t}$, where $n_{xx} = t_x^2$, $n_{xy} = n_{yx} = t_x t_y$, and $n_{yy} = t_y^2$. In order to reduce the effects of noise and image artifacts, the average of the nematic tensor was found in a small region, typically 40 x 40 or 80 x 80 pixels. The director was then found for each of these regions by computing the eigenvector with the largest eigenvalue of the corresponding average nematic tensor.

[1] S. J. DeCamp, G. S. Redner, A. Baskaran, M. F. Hagan, and Z. Dogic, Orientational order of motile defects in active nematics, Nature Materials **14**, 1110 (2015).

[2] M. H. Lee, C. P. Lapointe, D. H. Reich, K. J. Stebe, and R. L. Leheny, Interfacial hydrodynamic drag on nanowires embedded in thin oil films and protein layers, Langmuir **25**, 7976 (2009).

[3] W. Thielicke and E. J. Stamhuis, Pivlab - time-resolved digital particle image velocimetry tool for matlab (version: 2.00) (2014).

[4] A. Boudaoud, A. Burian, D. Borowska-Wykret, M. Uyttewaal, R. Wrzalik, D. Kwiatkowska, and O. Hamant, Fibriltool, an imagej plug-in to quantify fibrillar structures in raw microscopy images, Nature protocols **9**, 457 (2014).

[5] P. Bose, J. Eyckmans, T. D. Nguyen, C. S. Chen, and D. H. Reich, Effects of geometry on the mechanics and alignment of three-dimensional engineered microtissues, ACS Biomaterials Science & Engineering **5**, 3843 (2018).
